# Reversible gel–sol photoswitching with an overcrowded alkene-based bis-urea supergelator†

**DOI:** 10.1039/c6sc00659k

**Published:** 2016-03-22

**Authors:** Sander J. Wezenberg, Christelle M. Croisetu, Marc C. A. Stuart, Ben L. Feringa

**Affiliations:** a Stratingh Institute for Chemistry, University of Groningen Nijenborgh 4 9747 AG Groningen The Netherlands s.j.wezenberg@rug.nl b.l.feringa@rug.nl; b Groningen Biomolecular Sciences and Biotechnology Institute, University of Groningen Nijenborgh 7 9747 AG Groningen The Netherlands

## Abstract

A new type of low-molecular-weight gelator (LMWG), *i.e.* overcrowded alkene-based bis-ureas, can be switched effectively between *cis* and *trans* isomers using light as demonstrated by ^1^H NMR and UV-Vis spectroscopy. Gelation studies reveal that one of the synthesized *trans* compounds forms stable gels in aromatic hydrocarbon solvents down to a critical concentration of 0.4 mg mL^−1^. Transmission electron microscopy (TEM) shows that this gel consists of an entangled fibrous network. For the *trans* isomer of this LMWG intermolecular urea hydrogen bonding is observed in the solid state, whereas density functional theory (DFT) geometry optimization of the *cis* isomer indicates the possible formation of an intramolecular hydrogen bond. Irradiation of the gel triggers *trans*-to-*cis* isomerization and consequently, a gel–sol phase transition. This process can be fully reversed by altering the irradiation wavelength.

## Abstract 2

A low-molecular-weight organogel composed of photoresponsive bis-urea gelators can undergo a reversible gel–sol phase transition upon UV irradiation.

## Graphical abstract

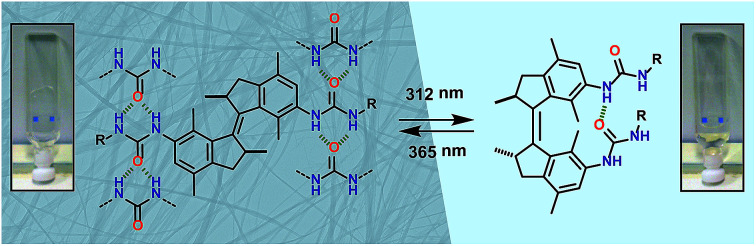

## Introduction

The interest in self-assembled supramolecular materials that can respond or adapt to external stimuli is rapidly expanding.^1^ This is not surprising when one considers the potential applications of these materials in important areas, for example, drug delivery,^2^ self-healing,^3^ and chemosensing.^4^ In particular, physical gels formed by low-molecular-weight gelators (LMWG) are highly versatile and effective.^5^ The ease of disrupting the weak non-covalent interactions between LMWGs is what makes these gels responsive to a wide variety of chemical and physical stimuli.^6^

Bis-urea compounds are known to be excellent LWMGs that can gelate a wide range of solvents at low concentrations.^7,8^ Dissolution of bis-urea gels has been successfully triggered by anion coordination,^9^ and mechanical force,^10^ among others. The use of light as a non-invasive stimulus to control gelation properties has attracted much attention because it can be delivered with high spatiotemporal precision and does not produce waste.^11^ For this reason, van Esch and Feringa developed photoresponsive bis-urea LMWGs having azobenzene and dithienylethene core structures.^12^ However, for the azobenzene bis-urea derivatives the photochemical isomerization was blocked in the gel state and for the dithienylethenes, photoswitching had to be performed at elevated temperature (105–110 °C) to be able to induce a gel–sol transition. These studies accentuate that it is still a major challenge to develop light-responsive LMWGs of which (i) photoswitching is not constrained in the gel state and (ii) of which the gelation behaviour is sufficiently altered in the photogenerated state for undergoing a gel–sol transition.

**Scheme 1:**
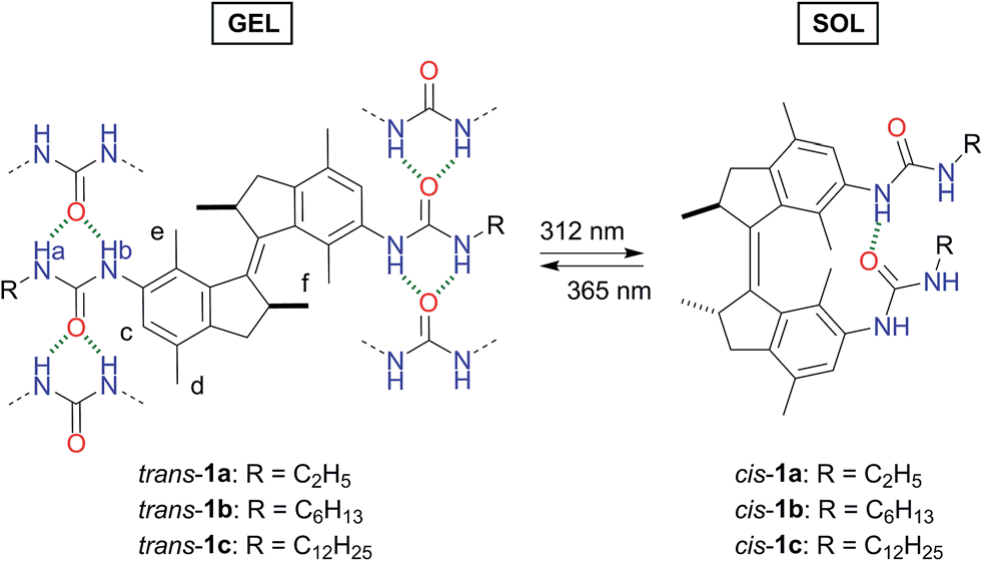
Photoisomerization behaviour and predicted urea hydrogen bonding pattern of *trans* and *cis* bis-urea LMWGs 1a–c.

Our group has developed various overcrowded alkene-based molecular switches,^13^ which have been applied to control, for example, magnetic and fluorophore interactions,^14^ anion binding,^15^ and aggregation^16^ by light. Yang and co-workers have recently functionalized a structurally related stiff stilbene switch with quadruple hydrogen bonding ureidopyrimidinone units.^11*e*^ Using light, they were able to alternate between supramolecular polymer and dimer formation. Irradiation of a concentrated polymer gel, or alternatively the addition of an acid, resulted in gel dissolution. Herein, we present the first overcrowded-alkene based bis-urea LMWGs 1a–c (Scheme 1) that can undergo a *trans*-to-*cis* stilbene-type isomerization in response to UV irradiation.

We found that the hexyl derivative *trans*-1b forms stable gels at very low concentrations (down to 0.4 mg mL^−1^) in aromatic hydrocarbons. Gel dissolution can be triggered by irradiation and is most likely due to the obstruction of intermolecular hydrogen bonding, which is necessary to form fibres, in the photogenerated *cis* form. This process is fully reversible and hence, light can be used to switch between gel and solution (sol) phase on demand. The results presented here pave the way for further development of robust photo-switchable bis-urea gels to be used in diverse nanotechnology applications.

## Results and discussion

### Synthesis and isomerisation behaviour

The urea end groups are known to have a large influence on the gelation properties.^7^ Therefore, the alkyl chain length was varied (*i.e.* ethyl, hexyl, dodecyl) in bis-urea LMWGs *trans*-1a–c. Reaction of the previously reported bis-amine precursor *trans*-S1 (ref. 17) with the respective isocyanate in CH_2_Cl_2_ at room temperature (see Scheme S1 in the ESI for details†) afforded the desired products *trans*-1a, b in good yield (80–90%) after filtration. The isolated yield of 1c was lower (55%) due to its sticky and glassy nature. The resulting bis-ureas, obtained and used as racemates, were fairly soluble in DMSO. Hence, their photoresponsive behaviour was studied in this solvent by ^1^H NMR and UV-Vis spectroscopy.

**Fig. 1:**
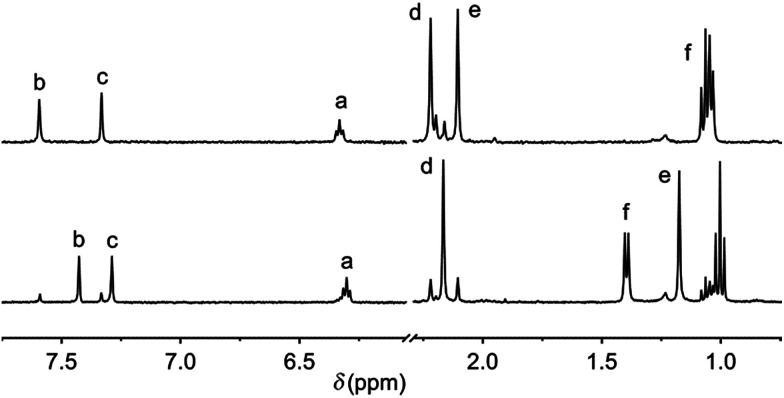
Aliphatic and aromatic region in the ^1^H NMR spectrum of 1a (1 mM in DMSO-*d*_6_) before (top) and after (bottom) 312 nm irradiation at 20 °C for 1 h. The top spectrum was recovered upon subsequent 365 nm irradiation for 1.5 h (see Fig. S6 in the ESI†). For the proton assignment, see Scheme 1.

Irradiation with 312 nm light of solutions of *trans*-1a–c, in DMSO-*d*_6_ induced ^1^H NMR spectral shifts that are indicative for formation of the *cis* isomer (Fig. 1 and S6–S8 in the ESI†).^13,15^ The *cis* : *trans* ratio at the photostationary state (PSS) was found to be 85 : 15 for each compound. Subsequent irradiation of the samples with 365 nm light led to full recovery of the ^1^H NMR spectrum of the starting *trans* isomer.

**Fig. 2:**
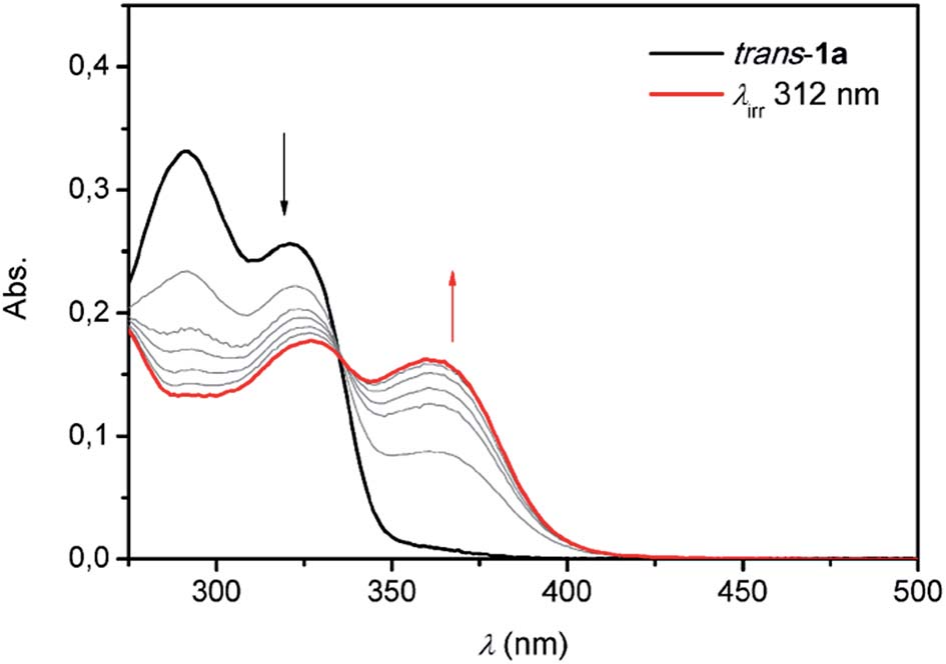
UV-Vis spectral changes upon irradiation with 312 nm light starting with *trans*-1a (black) at 1 × 10^−5^ M in DMSO and ending at the PSS (red).

By analogy with the *trans*-to-*cis* isomerisation of structurally related overcrowded alkenes,^13,15^ irradiation with 312 nm light of *trans*-1a–c resulted in a bathochromic shift of the UV-Vis absorption maxima (see Fig. 2 and S9–S11 in the ESI†). For bis-urea 1a for example, the absorption maxima at *λ* = 290 nm and 320 nm decreased and a new band appeared at *λ* = 360 nm. For all compounds, clear isosbestic points were observed which confirms that the photochemically induced *trans*-to-*cis* isomerization is a unimolecular process.^18^

### Gelation solvent screening

**Fig. 3:**
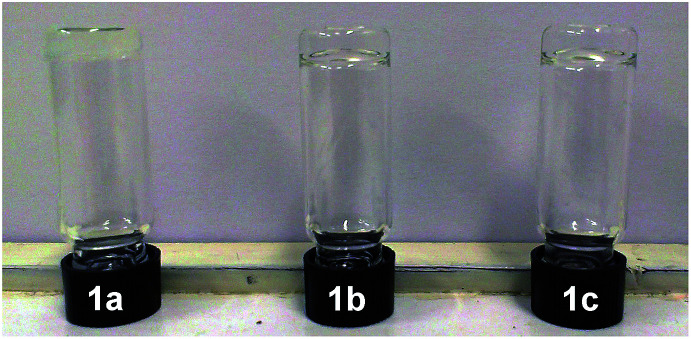
Gels obtained with *trans*-1a in CHCl_3_ and *trans*-1b, c in toluene at the critical gelation concentration (see Table 1).

The solubility and gelation properties of *trans*-1a–c were tested in a wide range of organic solvents (Table 1). A 10 mg mL^−1^ dispersion was repeatedly heated and sonicated to solubilise the material and the resulting solution was left to cool down to room temperature. In the case of poor solubility, more solvent was added to the sample. As can be observed in Table 1, the ethyl-appended *trans*-1a was insoluble in most of the selected solvents or it precipitated upon cooling of the solution. Nevertheless, an opaque gel was obtained within a few min of cooling from a CHCl_3_ solution as evidenced by the test tube inverting method (Fig. 3).

**Table 1:** Solubility and gelation properties of bis-ureas 1a–c^a^

Solvent	1a	1b	1c
DMSO	p	g/p	g/p
DMF	p	p	p
MeCN	i	i	i
EtOH	p^b^	p^b^	p
1,4-Dioxane	i	p	p
EtOAc	i	i	i
CHCl_3_	g (3 mg mL^−1^)	s	s
THF	i	p^c^	p^c^
Xylene	i	g (0.4 mg mL^−1^)	g (3 mg mL^−1^)
Toluene	i	g (0.4 mg mL^−1^)	g (4 mg mL^−1^)
Dodecane	i	i	i
Hexane	i	i	i

a Abbreviations g: gel (between brackets the critical gelation concentration), i: insoluble, p: precipitate, g/p: gel-like precipitate, s: soluble at 20 °C at 10 mg mL^−1^.

b Solubility in hot EtOH ≤ 2 mg mL^−1^.

c Solubility in hot THF ≤ 5 mg mL^−1^.

The bis-urea LMWGs *trans*-1b and *trans*-1c with the longer alkyl substituents (*i.e.* hexyl and dodecyl, respectively) were generally better soluble than *trans*-1a (Table 1). Transparent gels formed within a couple of min of cooling a solution in the aromatic solvents xylene and toluene (Fig. 3). It is really remarkable that the hexyl derivative (*trans*-1b) forms toluene and xylene gels down to concentrations as low as 0.4 mg mL^−1^, comparable to that of the earlier classified “supergelators”.^19^

**Fig. 4:**
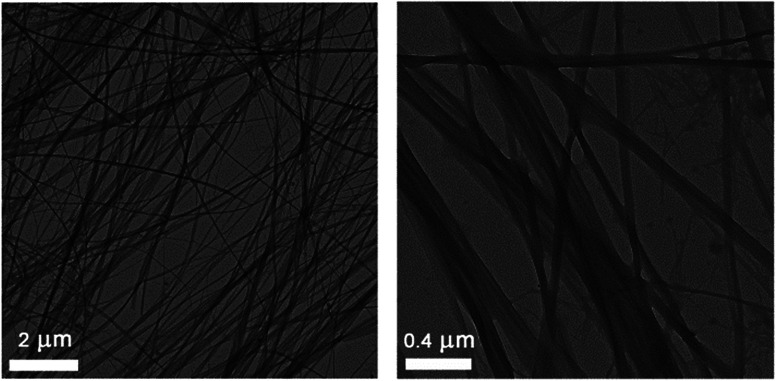
Electron micrographs of a toluene gel (0.5 mg mL^−1^) of *trans*-1b.

The morphology of the toluene gel obtained with the superior LMWG *trans*-1b was studied with transmission electron microscopy (TEM). Thread-like fibres were observed (Fig. 4), which fuse and intertwine to form an entangled network. The thicker fibres are bundles of thinner fibres and the smallest fibre diameter that could be measured is around 30 nm. Similar thread-like fibre formation has been reported for other bis-urea compounds.^8*a*,*b*^

### X-ray and DFT structural analysis

**Fig. 5:**
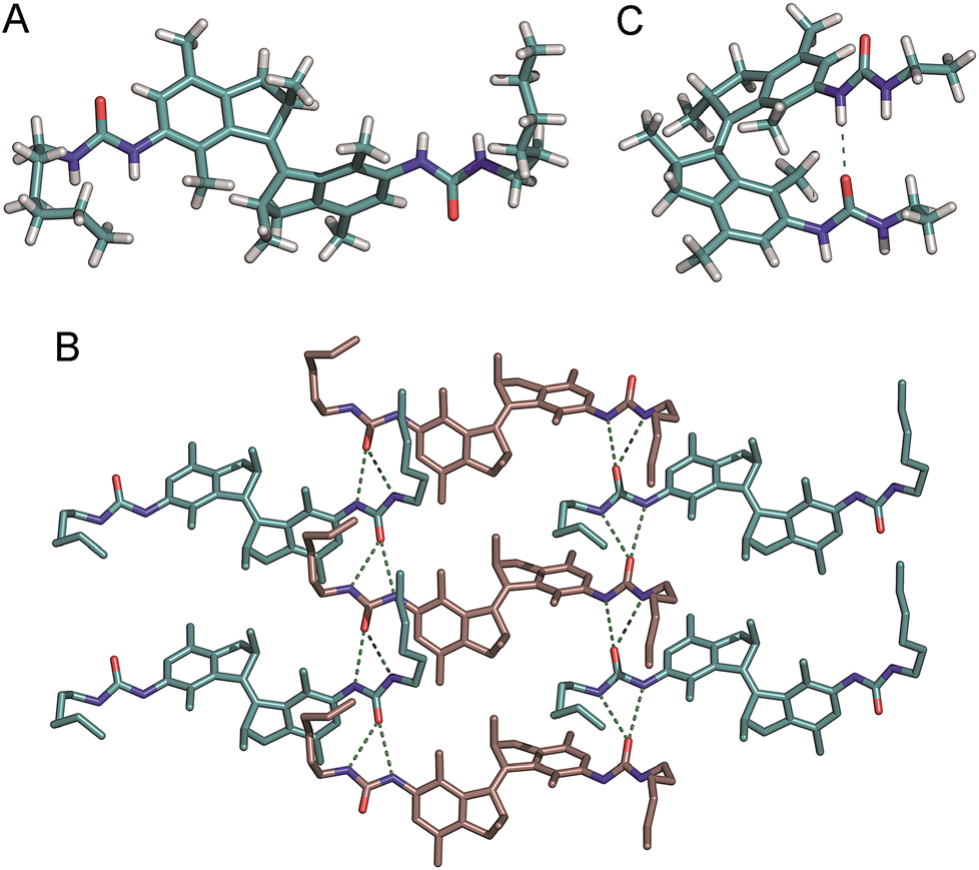
(A) X-ray molecular structure of *trans*-1b and (B) crystal packing showing the intermolecular hydrogen bond arrays. The (*R*,*R*)-enantiomer is shown in blue, the (*S*,*S*)-enantiomer in pink. Hydrogen atoms have been omitted in the packing for clarity. Selected hydrogen bond distances (Å): N⋯O: 3.053(2), 2.996(2), 2.940(2), 2.936(2). (C) DFT energy minimized structure [B3LYP 6-31G+(d,p), IEFPCM toluene] of (*R*,*R*)-*cis*-1a showing an intramolecular hydrogen bond. N⋯O distance: 3.216 Å.

Recrystallization of *trans*-1b from MeOH afforded single crystals that were suitable for X-ray crystallography (Fig. 5A and B). Solid state structures of other bis-urea LMWGs most often exhibit two antiparallel hydrogen bonded urea moieties between adjacent molecules.^8*a*,20^ The resulting formation of long urea hydrogen bonded linear chains is considered to be the reason for their universal gelation ability. In the crystal structure of *trans*-1b, however, each urea unit is linked to a different neighbour giving rise to a two dimensional network.^21^ Interestingly, the neighbouring molecules alternate in chirality between (*R*,*R*) and (*S*,*S*). Most likely, this hydrogen bond arrangement in the solid state is not representative for that in the gel state since fibres with high aspect ratio are usually formed *via* linear self-assembly.^20^ Furthermore, urea hydrogen bonding N⋯O distances are in the range of 2.9–3.1 Å, which is similar to those observed before for other bis-urea gelators.^20^

The structure of the photogenerated *cis* isomer was optimized by DFT calculations on the B3LYP/6-31G+(d,p) level of theory using an IEFPCM, toluene solvation model (see the ESI for details†). The energy minimized structure (Fig. 5C) reveals the possible formation of an intramolecular urea hydrogen bond with an N⋯O distance of 3.2 Å. Another possibility that cannot be excluded is self-complementary dimerization as was previously observed for an ureidopyrimidinone-functionalized stiff stilbene derivate.^11*e*^ Nevertheless, formation of intermolecular hydrogen bonding arrays as seen with the *trans* isomer, necessary to form fibres, is presumed to be unlikely for the *cis* isomer.

### Photocontrol of gel–sol transitions

**Fig. 6:**
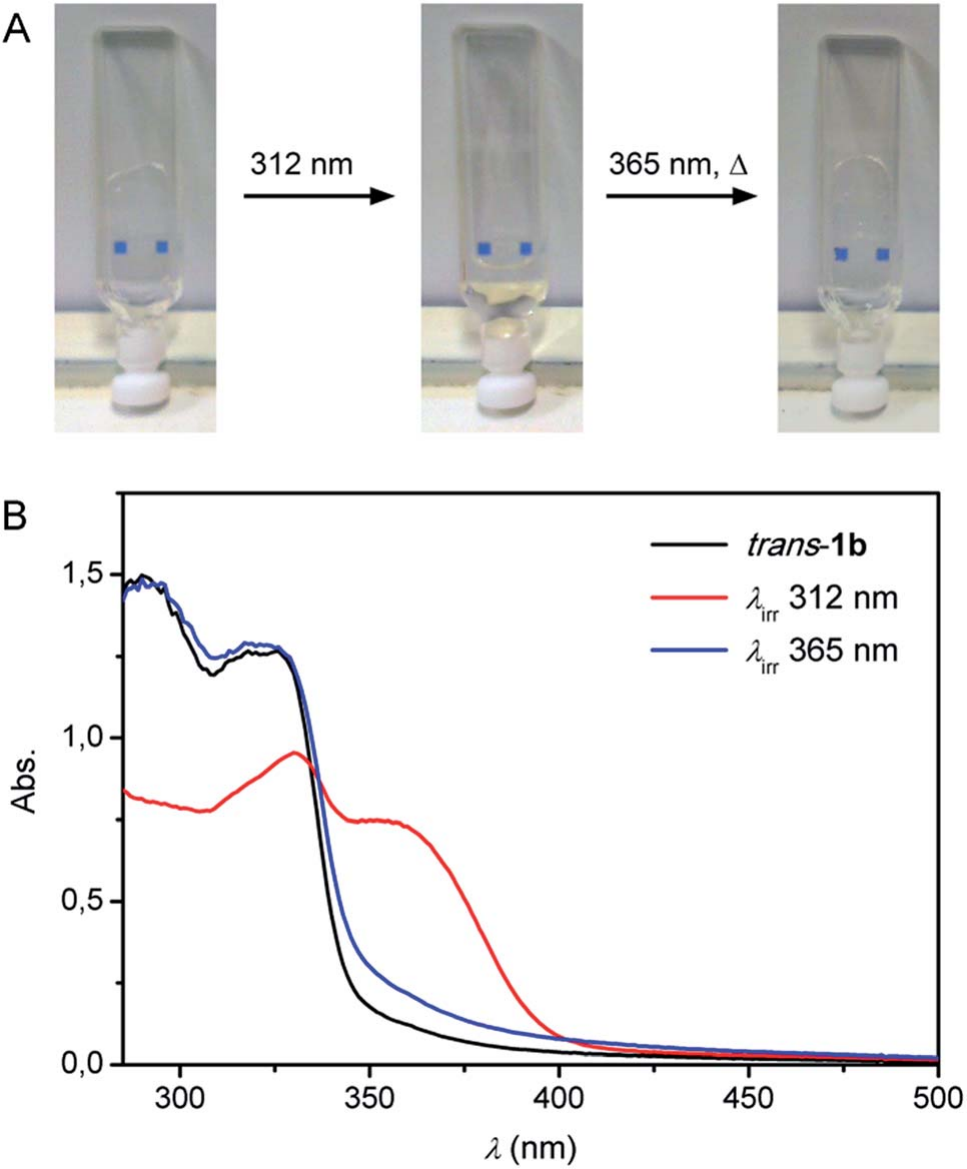
(A) 0.5 mg mL^−1^ toluene gel of *trans*-1b in a 1 mm quartz cuvette before and after 15 min irradiation with 312 nm light showing gel dissolution. The resulting solution was irradiated for 15 min with 365 nm light and gently heated to reform the gel upon slow cooling. (B) UV-Vis spectra measured during the gel–sol–gel switching cycle described above.

Structural analysis (*vide supra*) showed the formation of an intermolecular urea hydrogen bonded network for *trans*-1b and predicted intramolecular urea hydrogen bonding for the *cis* isomer. Based on these geometrical constraints, a poorer gelation ability was expected for *cis*-1b offering the possibility of photoinducing a gel–sol transition. To test this, a 0.5 mg mL^−1^ gel of *trans*-1b in toluene was transferred to a quartz cuvette and irradiated with 312 nm light for 15 min (Fig. 6A). Complete gel dissolution was observed and simultaneous recording of the UV-Vis spectrum (Fig. 6B), revealing a bathochromic shift, confirmed that *trans*-to-*cis* photoisomerization had taken place.^22^ Then, the obtained solution was irradiated for 15 min with 365 nm light followed by gentle heating.^23^ A transparent gel was again obtained upon cooling of the solution to room temperature proving the reversibility of this process.

Whereas TEM analysis displayed fibre formation for the *trans* isomer (see Fig. 4), no fibres could be observed anymore by analysing the same sample that was obtained after 312 nm light irradiation (Fig. S17 in the ESI†). This result supports our hypothesis that fibre formation *via* intermolecular hydrogen urea bonding is blocked in the photogenerated *cis* state. On the other hand, when an identical sample was dried and analysed by FT-IR spectroscopy, absorptions characteristic for hydrogen bonded ureas were found (*i.e.* 3321 cm^−1^, 1644 cm^−1^ and 1558 cm^−1^ for the NH stretch, amide I and amide II vibrations, respectively, Fig. S18 in the ESI†).^24^ These IR absorptions do not differ significantly from those observed for *trans*-1b and suggest that the *cis* isomer is capable of urea hydrogen bonding.

## Conclusions

In summary, we have described a new set of photoresponsive bis-urea LMWGs based on an overcrowded alkene switch. The hexyl-urea derivative *trans*-1b gelates aromatic hydrocarbons down to a critical concentration of 0.4 mg mL^−1^ and can therefore be categorized as “supergelator”. In the photogenerated *cis* state intermolecular hydrogen bonds are much less likely to form and hence, gel dissolution occurs upon the photoinduced *trans*-to-*cis* isomerization. Interestingly, this process can be reversed by light-irradiation to reobtain the gel phase. To the best of our knowledge, this work represents the most effective strategy towards reversible photoswitching of low-molecular-weight bis-urea gels. Therefore, this system is potentially suitable for functional applications such as self-healing and drug delivery. Current efforts in our lab are targeted at producing light-responsive hydrogels and exploitation of the unique chirality of these systems.

## Experimental section

### General methods and materials

Toluene and CH_2_Cl_2_ were dried by using an MBraun solvent purification system and solvents were degassed by purging with N_2_ for 30 min. The bis-amine precursor *trans*-S1 (see Scheme S1 in the ESI†) was prepared according to a procedure described in the literature.^17^ All other chemicals were commercial products and were used as received. Melting points (mp) were determined using a Büchi-B545 capillary melting point apparatus. ^1^H and ^13^C NMR spectra were recorded on Varian Mercury Plus-400 and Agilent 400-MR spectrometers at 298 K. Chemical shifts (*δ*) are denoted in parts per million (ppm) relative to DMSO-*d*_6_ (*δ*_H_ 2.50 ppm) or CDCl_3_ (*δ*_C_ 77.16 ppm). For ^1^H NMR spectroscopy, the splitting pattern of peaks is designated as: s (singlet), d (doublet), t (triplet), m (multiplet), or br (broad). High-resolution mass spectrometry (ESI-MS) was performed on a LTQ Orbitrap XL spectrometer with ESI ionization. UV-Vis absorption spectra were recorded on a Hewlet-Packard HP 8543 diode array with a Peltier heating/cooling element. IR spectra were measured on a Perkin Elmer Spectrum 400 FT-IR instrument and the bands are listed from 4000 to 600 cm^−1^ with intensities: s, m, w, br for broad and sh for shoulder. UV-Vis and ^1^H NMR irradiation experiments were carried out using a Spectroline model ENB-280C/FE lamp positioned at a distance of 3 cm from the sample of which the temperature was maintained at 20 °C.

### General procedure for the synthesis of bis-urea LMWGs

The respective alkyl isocyanate (0.40 mmol) was added to the bis-amine precursor *trans*-S1 (0.20 mmol) in CH_2_Cl_2_ (4 mL) under a N_2_ atmosphere. The solution was stirred for 16 h and the white precipitate was filtered off and washed with CH_2_Cl_2_ to afford bis-urea LMWGs *trans*-1a–c as white solids.

### Bis-ethylurea *trans*-1a

Yield: 90%. Mp 261 °C; FT-IR (ATR) *ν*_max_/cm^−1^ 3327 (m, br), 2968 (m), 2928 (m), 2868 (w, sh), 1643 (s), 1547 (s), 1453 (m), 1249 (s), 1211 (m, sh), 1058 (w), 871 (w, br), 772 (w), 754 (w, sh); ^1^H NMR (400 MHz, DMSO-*d*_6_, assignment based on COSY) *δ*_H_ 7.58 (s, 2H; NH), 7.33 (s, 2H; ArH), 6.32 (t, *J* = 5.5 Hz, 2H; NH), 3.15–3.08 (m, 6H; CH, CH_2_), 2.79–2.72 (m, 2H; CH), 2.22 (s, 6H; ArCH_3_), 2.18 (d, *J* = 14.4 Hz, 2H; CH), 2.11 (s, 6H; ArCH_3_), 1.09–1.03 (m, 12H; CH_3_, ArCH_3_); too insoluble for ^13^C NMR measurement; HRMS (ESI) *m*/*z*: 489.3209 ([M + H]^+^, calcd for C_30_H_41_N_4_O_2_^+^: 489.3224).

### Bis-hexylurea *trans*-1b

Yield: 82%. Mp 242 °C; FT-IR (ATR) *ν*_max_/cm^−1^ 3325 (m, br), 2953 (m, sh), 2925 (m), 2856 (m), 1643 (s), 1555 (s), 1453 (m), 1343 (w), 1251 (s), 1212 (m, sh), 863 (w), 771 (w), 754 (w, sh); ^1^H NMR (400 MHz, DMSO-*d*_6_) *δ*_H_ 7.59 (s, 2H; NH), 7.35 (s, 2H; ArH), 6.35 (t, *J* = 5.6 Hz, 2H; NH), 3.11–3.05 (m, 6H; CH, CH_2_), 2.77–2.72 (m, 2H; CH), 2.22 (s, 6H; ArCH_3_), 2.18 (d, *J* = 14.4 Hz, 2H; CH), 2.10 (s, 6H; ArCH_3_), 1.47–1.39 (m, 4H; CH_2_), 1.33–1.26 (br, m, 12H; CH_2_), 1.04 (d, *J* = 6.4 Hz, 6H; CH_3_), 0.88 (t, *J* = 6.8 Hz, 6H; CH_3_); ^13^C NMR (100 MHz, CDCl_3_) *δ*_C_ 157.1, 142.8, 142.0, 141.1, 134.6, 132.8, 128.0, 126.5, 42.4, 40.6, 39.0, 31.7, 30.4, 26.7, 22.7, 19.2, 18.4, 18.1, 14.2; HRMS (ESI) *m*/*z*: 601.4459 ([M + H]^+^, calcd for C_38_H_57_N_4_O_2_^+^: 601.4476), 599.4306 ([M − H_2_ + H]^+^, calcd for C_38_H_55_N_4_O_2_^+^: 599.4320).

### Bis-dodecylurea *trans*-1c

Yield: 55%. Mp 249 °C; FT-IR (ATR) *ν*_max_/cm^−1^ 3332 (m, br), 2921 (s), 2851 (m), 1640 (s), 1555 (s), 1454 (m), 1371 (w), 1252 (s), 1212 (m, sh), 1072 (w), 865 (w, br), 773 (w), 754 (w, sh); ^1^H NMR (400 MHz, DMSO-*d*_6_) *δ*_H_ 7.57 (s, 2H; NH), 7.33 (s, 2H; ArH), 6.32 (t, *J* = 5.6 Hz, 2H; NH), 3.11–3.05 (m, 6H; CH, CH_2_), 2.78–2.72 (m, 2H; CH), 2.22 (s, 6H; ArCH_3_), 2.18 (d, *J* = 14.4 Hz, 2H; CH), 2.11 (s, 6H; ArCH_3_), 1.47–1.40 (br, m, 4H; CH_2_), 1.32–1.23 (br, m, 36H; CH_2_), 1.04 (d, *J* = 6.3 Hz, 6H; CH_3_), 0.85 (t, *J* = 6.4 Hz, 6H; CH_3_); ^13^C NMR (100 MHz, CDCl_3_) *δ*_C_ 157.1, 142.8, 141.9, 141.0, 134.6, 132.7, 127.9, 126.5, 42.4, 40.6, 39.0, 32.1, 30.4, 29.8 (4×), 29.5 (2×), 27.1, 22.8, 19.2, 18.4, 18.1, 14.3; HRMS (ESI) *m*/*z*: 769.6341 ([M + H]^+^, calcd for C_50_H_81_N_4_O_2_^+^: 769.6354), 767.6191 ([M − H_2_ + H]^+^, calcd for C_50_H_79_N_4_O_2_^+^: 767.6198).

### Gelation experiments

The compound of interest (1 mg) was dispersed in 0.1 mL of solvent in a closed vial. The vial was repetitively sonicated and heated using a heating gun until the solid had dissolved. In the case of poor solubility, additional solvent was added to the mixture. The solution was allowed to cool to room temperature and gelation was considered to have occurred when there was no gravitational flow upon inverting the vial.

### Gel–sol photoswitching experiments

A 0.5 mg mL^−1^ toluene gel of *trans*-1b was heated to give a solution and transferred to a 1 mm quartz cuvette. Gentle heating was followed by cooling to room temperature to reobtain the gel phase. Then the cuvette was placed upside down and irradiated with 312 nm light for 15 min using a Spectroline model ENB-280C/FE lamp positioned at a distance of 5 cm. The resulting solution was irradiated with 365 nm light for 15 min using the same lamp and slightly heated to reobtain the gel upon cooling to room temperature. After each step a UV-Vis absorption spectrum was recorded. In a separate experiment, an identically prepared 312 nm light irradiated sample was concentrated and an FT-IR (ATR) spectrum was measured.

### Transmission electron microscopy

A toluene gel of *trans*-1b was prepared as described above and placed on a carbon coated copper grid. Images were recorded on a FEI Technai T20 microscope at 200 kV with a slow scan CCD camera.

## Supplementary Material

SC-007-C6SC00659K-s001

SC-007-C6SC00659K-s002
