# Supplementary material for: Reversible gel–sol photoswitching with an overcrowded alkene-based bis-urea supergelator
Source: Chem Sci. 2016 Mar 22;7(7):4341–6. doi: 10.1039/c6sc00659k (PMC6013809; doi:10.1039/c6sc00659k)
Supplement: SC-007-C6SC00659K-s001 [file SC-007-C6SC00659K-s001.pdf]

Electronic Supplementary Material (ESI) for Chemical Science.
This journal is © The Royal Society of Chemistry 2016

Chemical Science

Electronic Supplementary Information (ESI)

Reversible gel-sol photoswitching with an overcrowded alkene-based bis-urea supergelator

Sander J. Wezenberg,*[a] Christelle M. Croisetu,[a] Marc C. A. Stuart[a,b] and Ben L. Feringa*[a]

[a] Stratingh Institute for Chemistry, University of Groningen, Nijenborgh 4, 9747 AG, Groningen, The Netherlands. Email: s.j.wezenberg@rug.nl, b.l.feringa@rug.nl

[b] Groningen Biomolecular Sciences and Biotechnology Institute, University of Groningen, Nijenborgh 7, 9747 AG, Groningen, The Netherlands.

Table of contents

Scheme of synthesis ESI-3

^{1}H and ^{13}C NMR Spectra of new compounds ESI-4

^{1}H NMR photochemical switching experiments ESI-7

UV-Vis photochemical switching experiments ESI-10

UV-Vis thermal stability experiments ESI-12

Single crystal X-ray analysis ESI-14

DFT Calculations ESI-15

Gel-sol photoswitching experiment ESI-18

Notes and references ESI-20

Scheme of synthesis

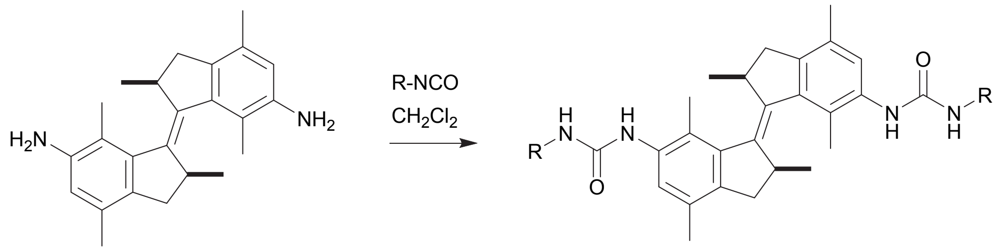

trans-**S1**

trans-**1a**: R = C_2H_5 (90%)
trans-**1b**: R = C_6H_{13} (82%)
trans-**1c**: R = $C_{12}H_{25}$ (55%)

Scheme S1 Synthesis of bis-urea LMWGs *trans*-**1a-c** starting from bis-amine *trans*-**S1**.

^{1}H and ^{13}C NMR Spectra of new compounds

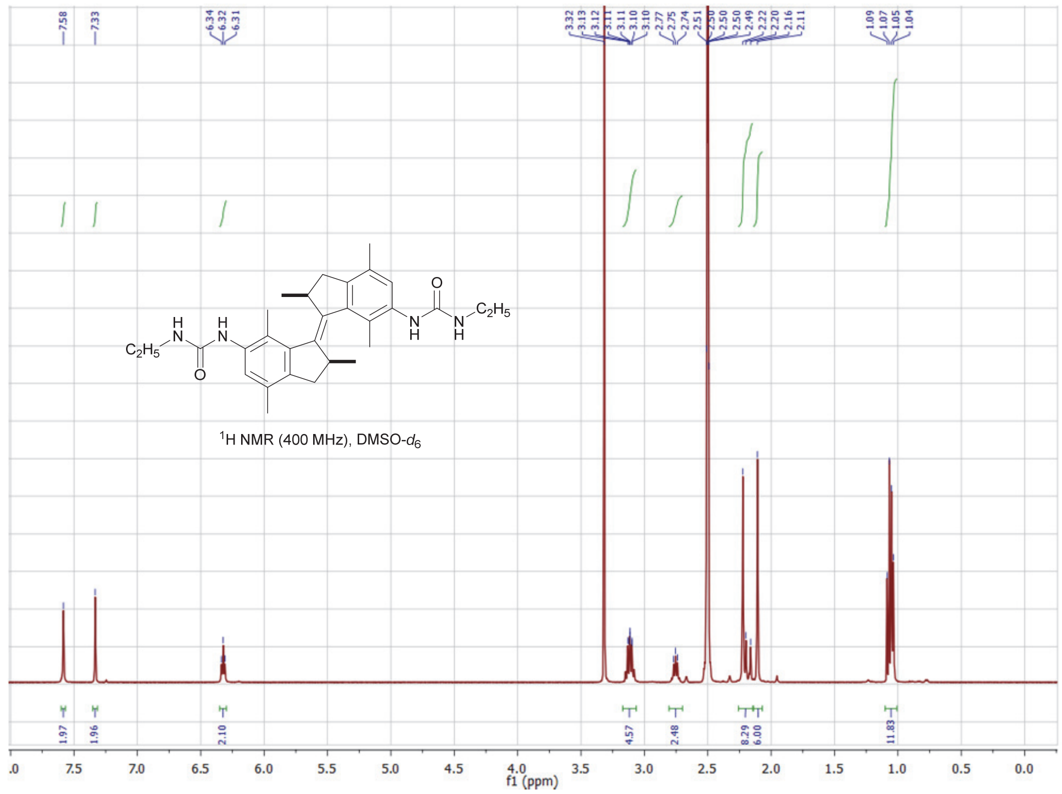

Fig. S1 ^{1}H NMR spectrum of *trans*-**1a** measured in DMSO-d_6 at 298 K. The compound was too insoluble for a proper ^{13}C NMR measurement.

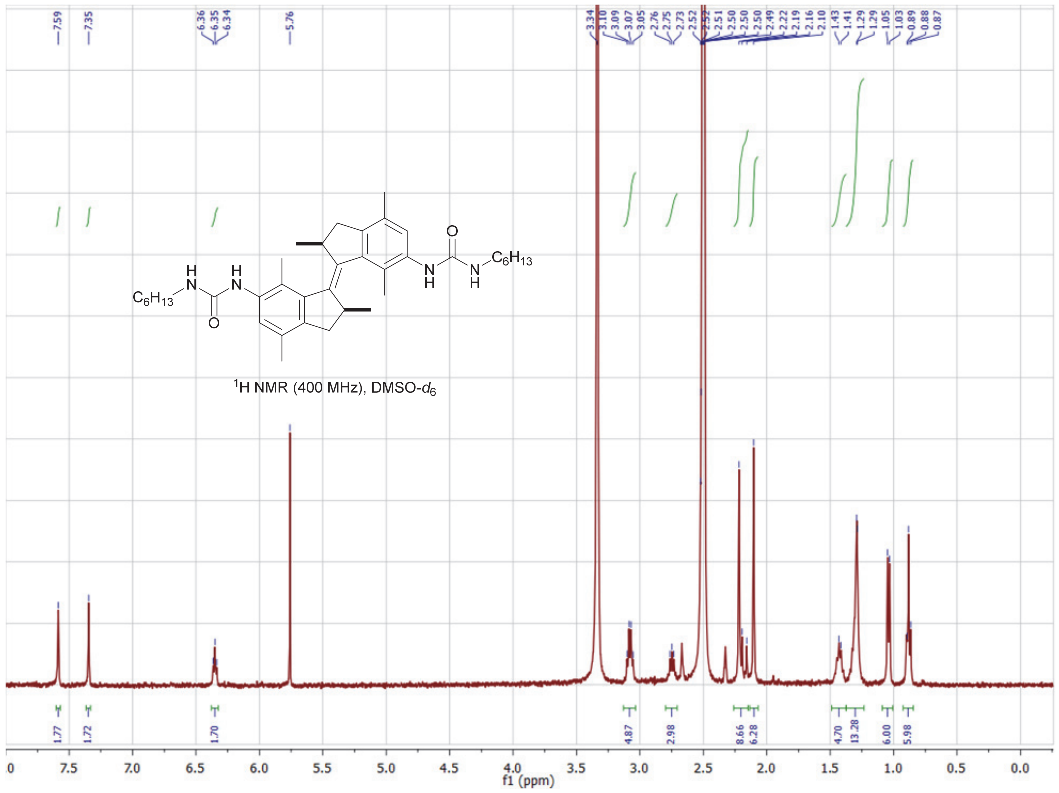

Fig. S2 ^{1}H NMR spectrum of *trans*-**1b** measured in DMSO-d_6 at 298 K.

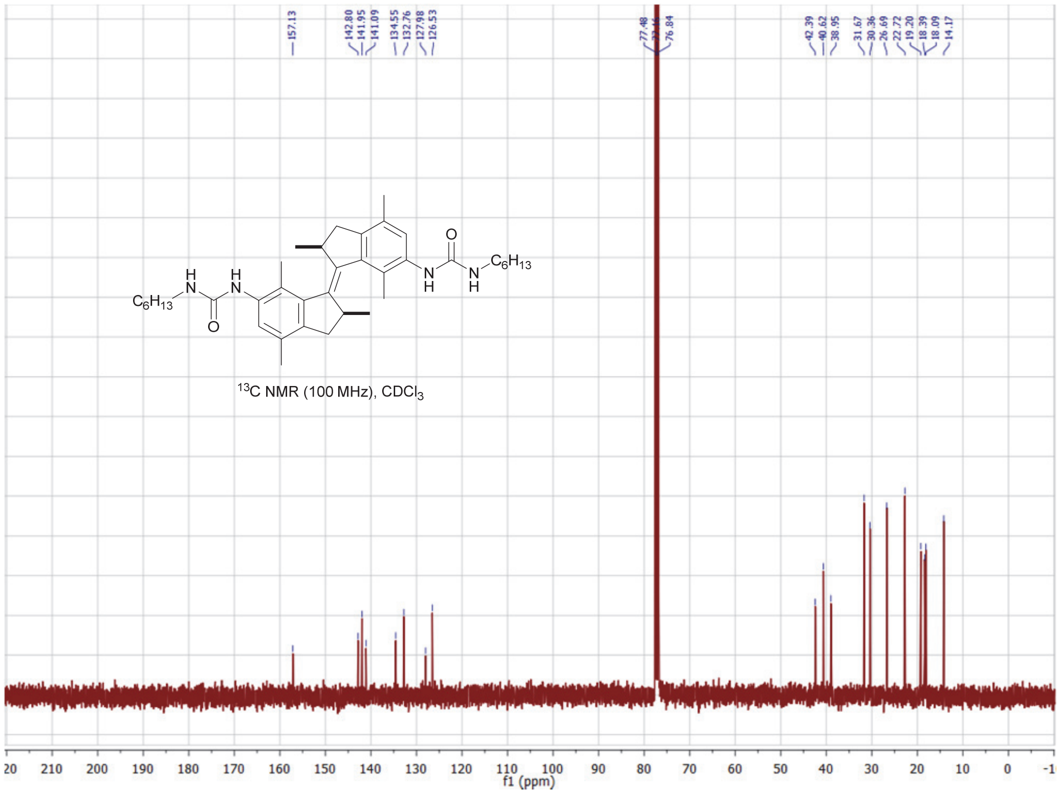

Fig. S3 ^{13}C NMR spectrum of *trans*-**1b** measured in $CDCl_3$ at 298 K.

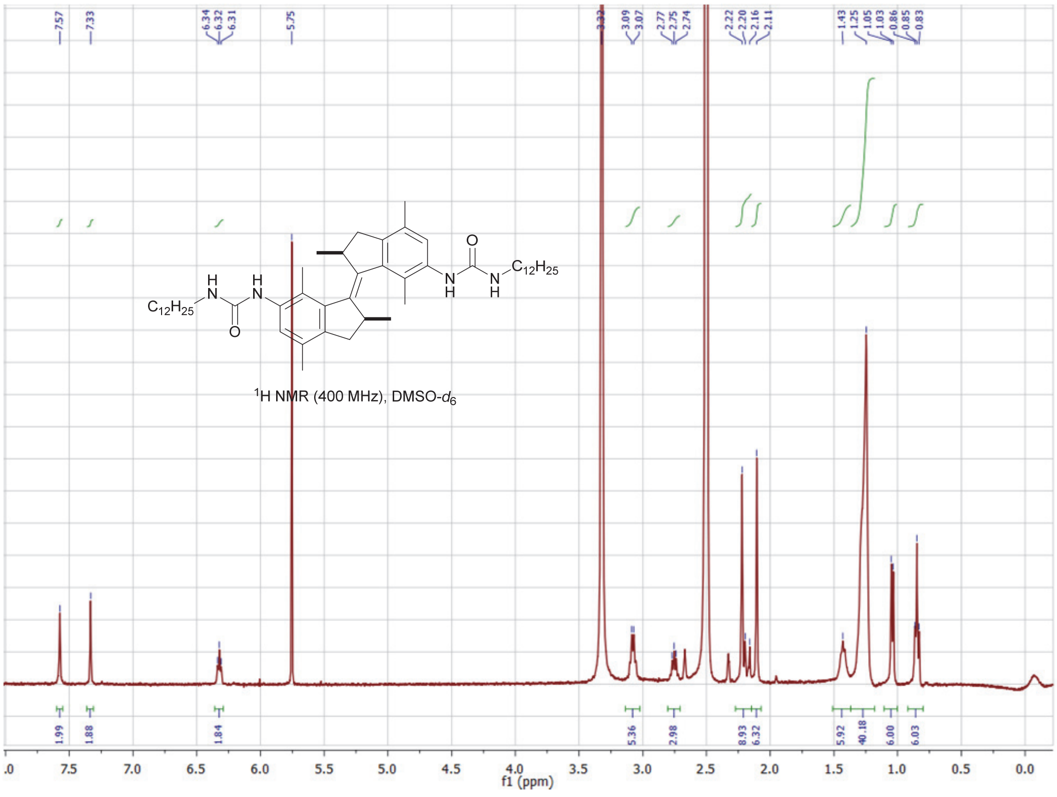

Fig. S4 ^{1}H NMR spectrum of *trans*-**1c** measured in DMSO-d_6 at 298 K.

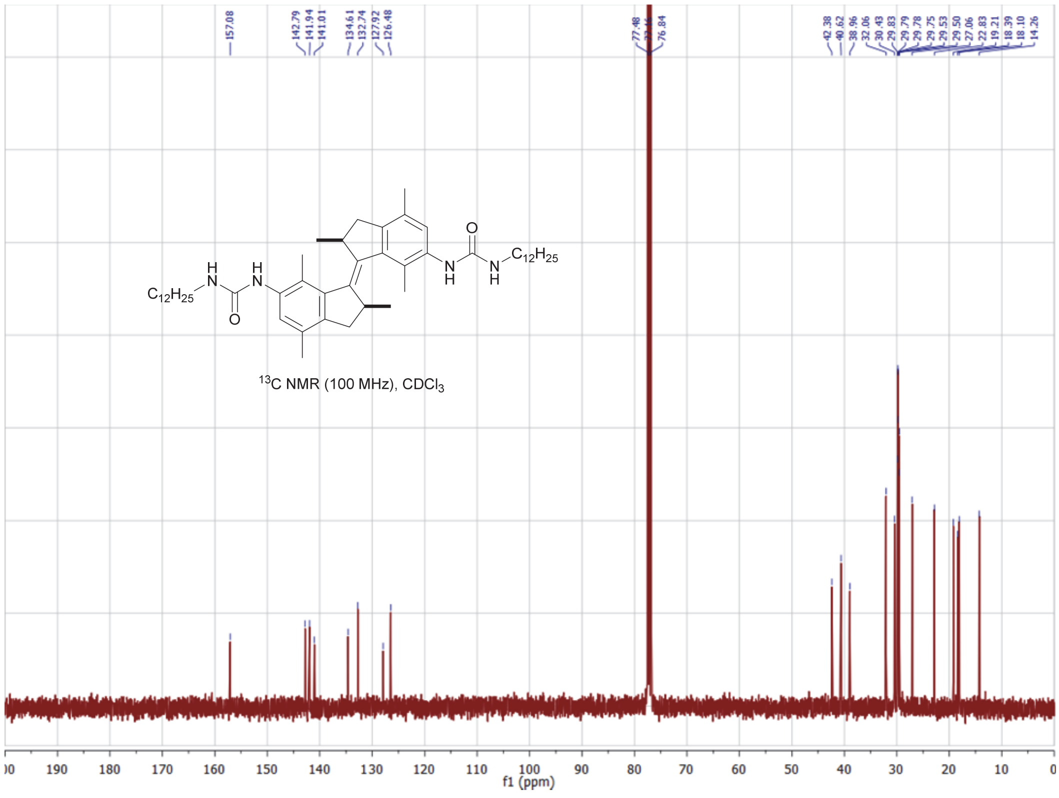

Fig. S5 ^{13}C NMR spectrum of *trans*-**1c** measured in $CDCl_3$ at 298 K.

^{1}H NMR photochemical switching experiments

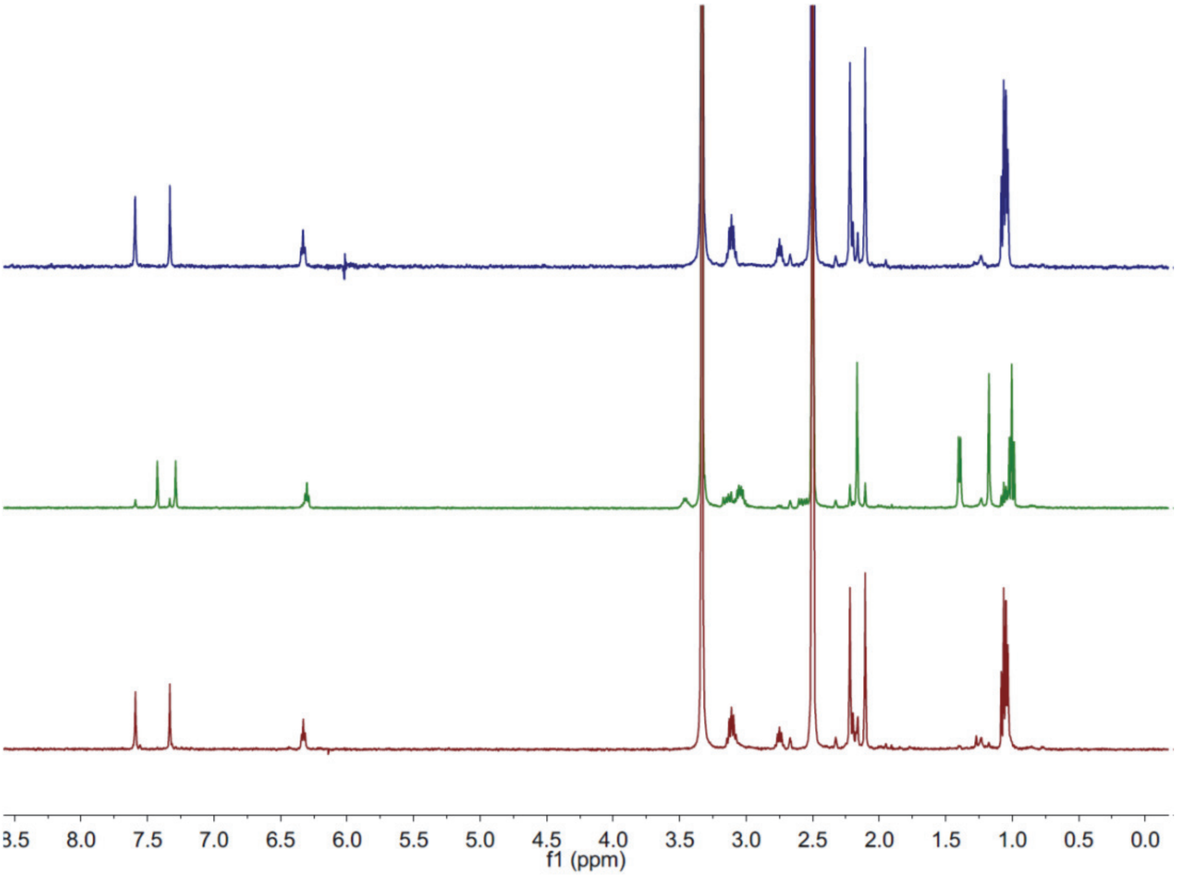

Fig. S6 ^{1}H NMR spectral changes upon photoirradiation of **1a** (1 mM in DMSO-d_6 at 298 K) starting with the *trans* isomer (blue) followed by irradiation with λ_{max} = 312 nm light at 20 °C for 1h to give the *cis* isomer (green) and subsequent irradiation with λ_{max} = 365 nm light for 1.5 h at 20 °C to regenerate the *trans* isomer (red). For the PSS determination, the average integral values of the urea NH and aromatic CH singlets was used.

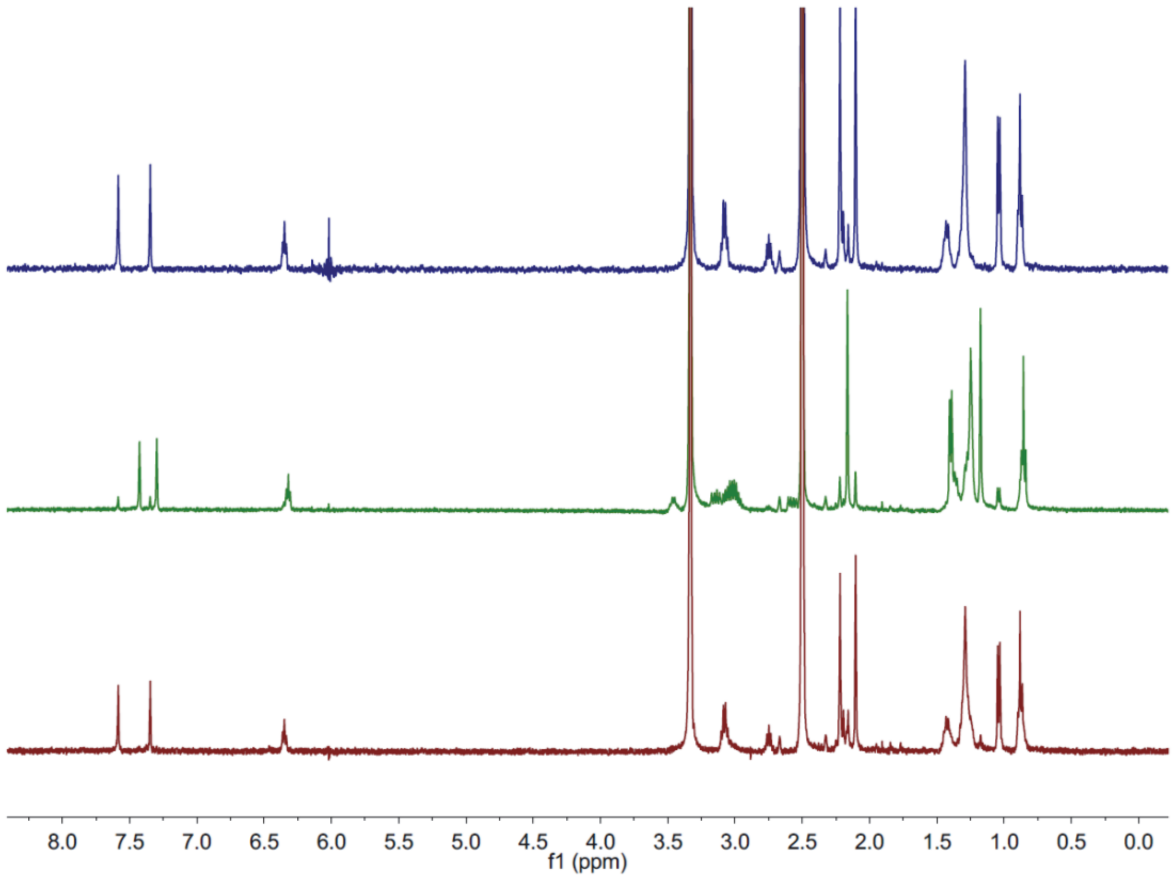

Fig. S7 ^{1}H NMR spectral changes upon photoirradiation of **1b** (1 mM in DMSO-d_6 at 298 K) starting with the *trans* isomer (blue) followed by irradiation with λ_{max} = 312 nm light at 20 °C for 1h to give the *cis* isomer (green) and subsequent irradiation with λ_{max} = 365 nm light for 1.5 h at 20 °C to regenerate the *trans* isomer (red). For the PSS determination, the average integral values of the urea NH and aromatic CH singlets was used.

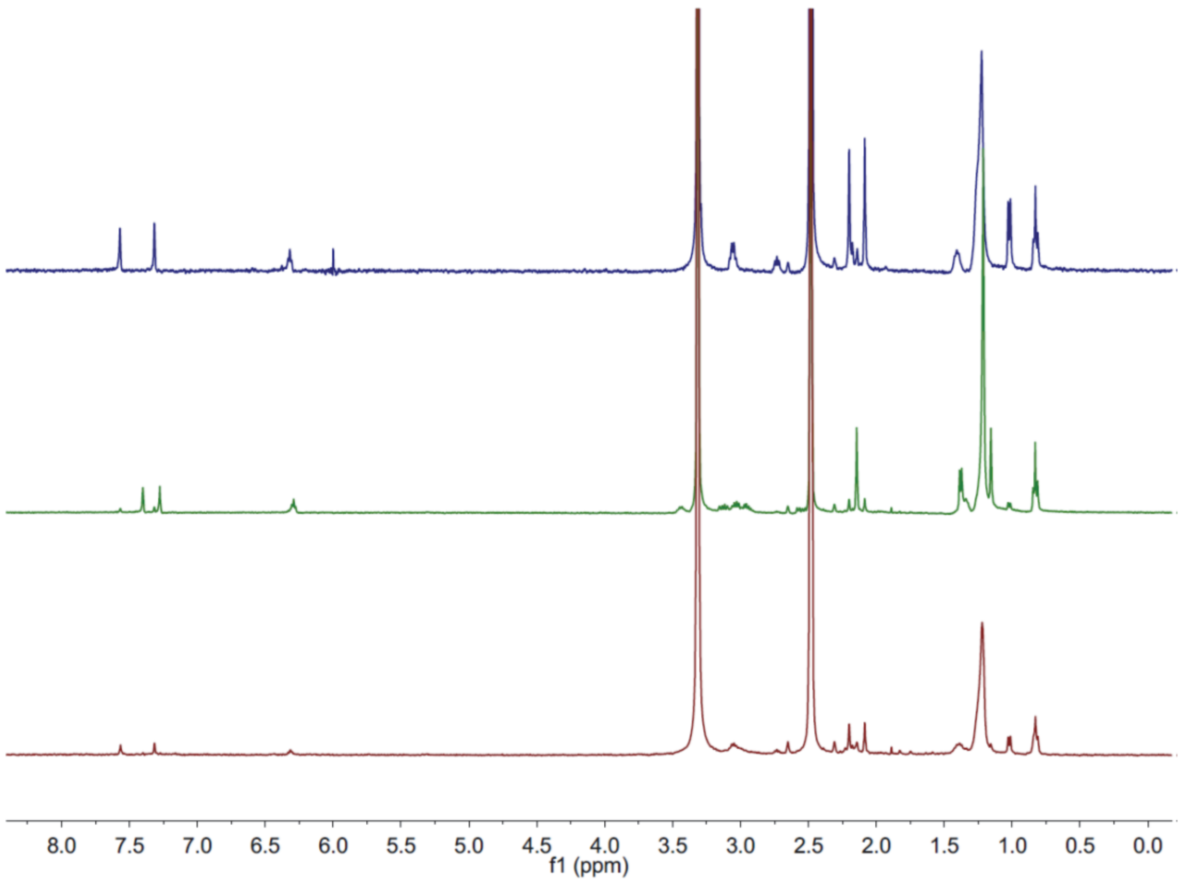

Fig. S8 ^{1}H NMR spectral changes upon photoirradiation of **1c** (1 mM in DMSO-d_6 at 298 K) starting with the *trans* isomer (blue) followed by irradiation with λ_{max} = 312 nm light at 20 °C for 1h to give the *cis* isomer (green) and subsequent irradiation with λ_{max} = 365 nm light for 1.5 h at 20 °C to regenerate the *trans* isomer (red). For the PSS determination, the average integral values of the urea NH and aromatic CH singlets was used.

UV-Vis photochemical switching experiments

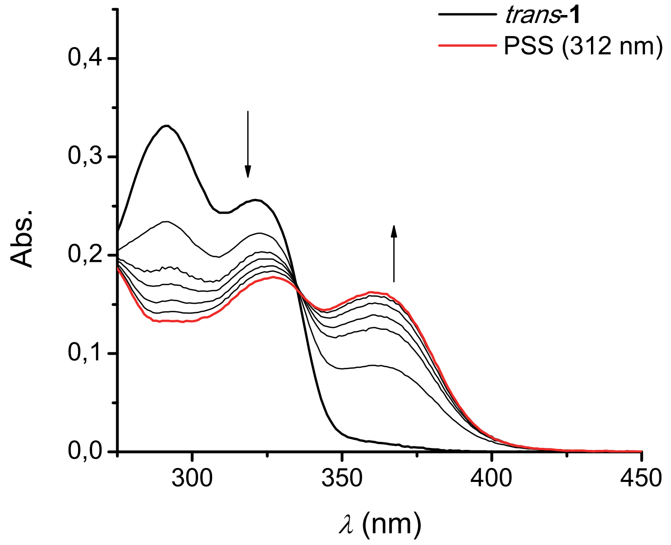

Fig. S9 UV-Vis absorption spectral changes starting with *trans*-**1a** (1×10^{-5} M in DMSO, black spectrum) upon irradiation with λ_{max} = 312 nm light at 20 °C for 5, 10, 15, 20, 25 and 30 minutes after which the photostationary state (PSS) was reached (red spectrum).

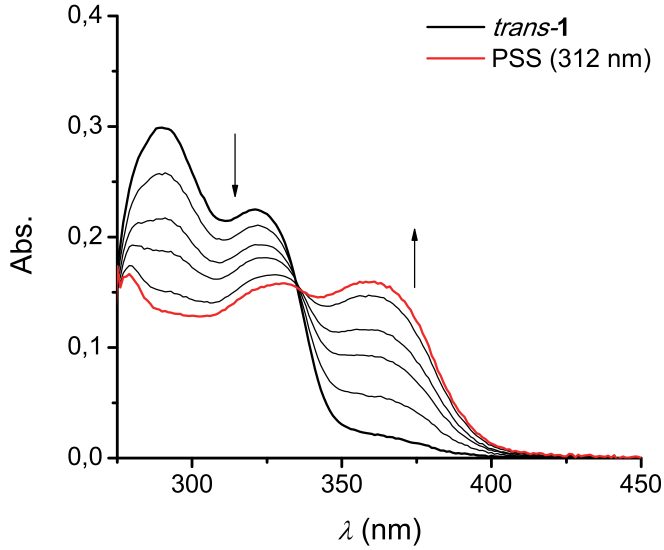

Fig. S10 UV-Vis absorption spectral changes starting with *trans*-**1b** (1×10^{-5} M in DMSO, black spectrum) upon irradiation with λ_{max} = 312 nm light at 20 °C for 5, 10, 15, 25 and 35 minutes after which the photostationary state (PSS) was reached (red spectrum).

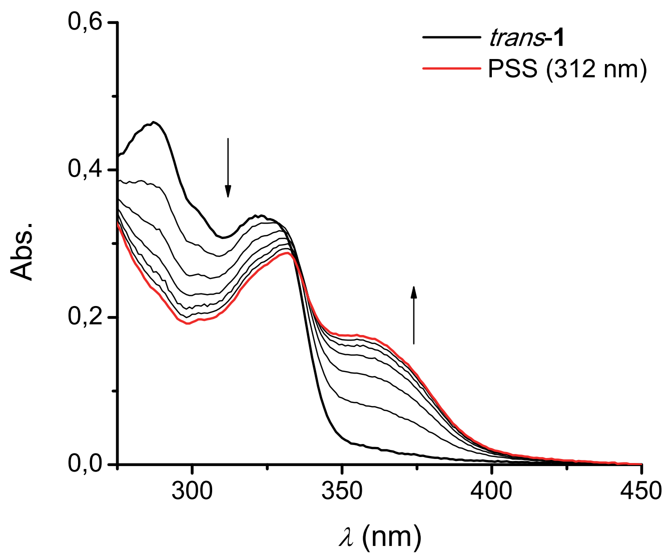

Fig. S11 UV-Vis absorption spectral changes starting with *trans*-**1c** (2×10^{-5} M in DMSO, black spectrum) upon irradiation with λ_{max} = 312 nm light at 20 °C for 5, 10, 15, 20, 25 and 30 minutes after which the photostationary state (PSS) was reached (red spectrum).

UV-Vis thermal stability experiments

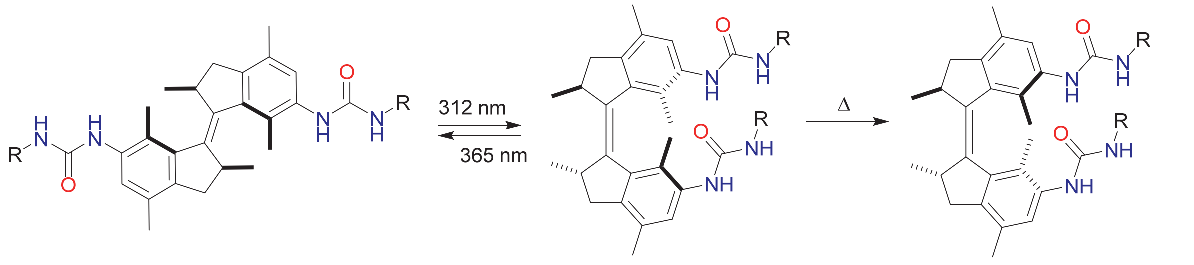

trans stable *cis* metastable *cis* stable

Scheme S2 Photochemical and thermal isomerization behaviour of bis-urea LMWGs **1a-c**. While irradiation with λ_{max} = 365 nm light of the metastable *cis* isomer leads to regeneration of stable *trans*, heating of the sample causes equilibration to a more stable *cis* form. This is accompanied by a hypsochromic shift in the UV-Vis spectrum.[1] The rate constants (k) for this process were determined at four temperatures (55, 60, 65, 70 °C) by monitoring the absorption decrease at 365 nm and fitting to the equation: $A = A_0e^{-kt}$ using Origin software.

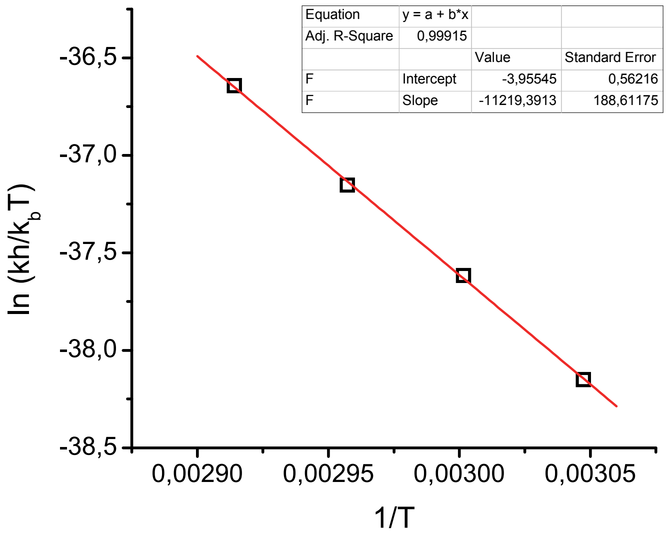

Fig. S12 Eyring plot analysis of the thermal isomerization step from metastable *cis*-**1a** to the more stable *cis*-**1a** monitored by the decrease in absorption at 365 nm in DMSO. $\Delta^{\ddagger}H$ = 93.3 kJ mol^{-1}; $\Delta^{\ddagger}S$ = –32.9 J mol^{-1} K^{-1}; $\Delta^{\ddagger}G$ (20 °C) = 102.9 kJ mol^{-1}; $t_{1/2}$ (20 °C) = 70 h.

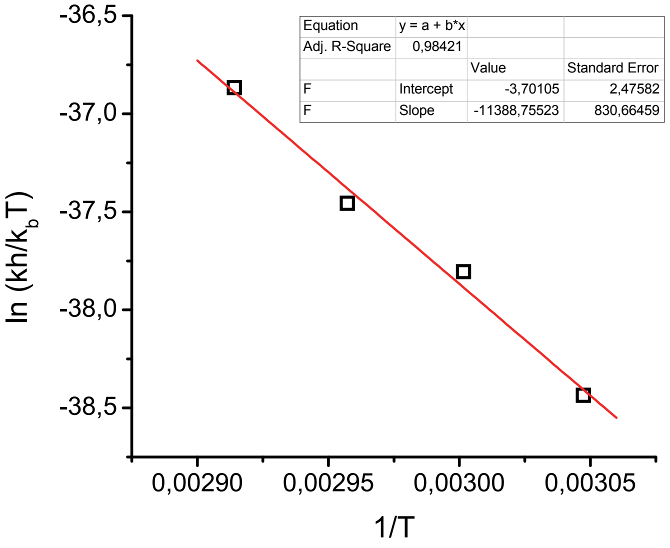

Fig. S13 Eyring plot analysis of the thermal isomerization step from metastable *cis*-**1b** to the more stable *cis*-**1b** monitored by the decrease in absorption at 365 nm in DMSO. $\Delta^{\ddagger}H = 94.7$ kJ mol^{-1}; $\Delta^{\ddagger}S = -30.8$ J mol^{-1} K^{-1}; $\Delta^{\ddagger}G$ (20 °C) = 103.7 kJ mol^{-1}; $t_{1/2}$ (20 °C) = 96 h.

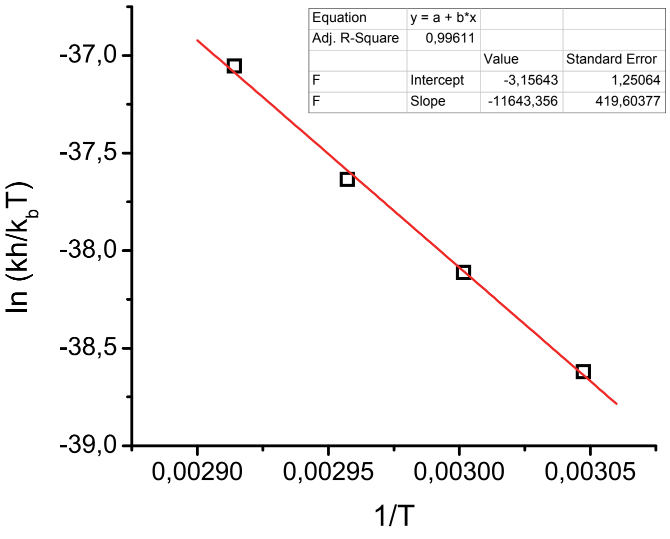

Fig. S14 Eyring plot analysis of the thermal isomerization step from metastable *cis*-**1c** to the more stable *cis*-**1c** monitored by the decrease in absorption at 365 nm in DMSO. $\Delta^{\ddagger}H = 96.8$ kJ mol^{-1}; $\Delta^{\ddagger}S = -26.2$ J mol^{-1} K^{-1}; $\Delta^{\ddagger}G$ (20 °C) = 104.5 kJ mol^{-1}; $t_{1/2}$ (20 °C) = 133 h.

Single crystal X-ray analysis

Single crystals of *trans*-**1b** were grown upon cooling of a solution in hot MeOH. A single crystal was mounted on top of a cryoloop and transferred into the cold nitrogen stream (100 K) of a Bruker-AXS D8 Venture diffractometer. Data collection and reduction was done using the Bruker software suite APEX2.[2] The final unit cell was obtained from the xyz centroids of 5420 reflections after integration. A multiscan absorption correction was applied, based on the intensities of symmetry-related reflections measured at different angular settings (*SADABS*). The structures were solved by direct methods using *SHELXT*,[3] and refinement of the structure was performed using *SHLELXL*.[4] The hydrogen atoms were generated by geometrical considerations, constrained to idealised geometries and allowed to ride on their carrier atoms with an isotropic displacement parameter related to the equivalent displacement parameter of their carrier atoms. Crystallographic data: $C_{38}H_{56}N_4O_2$; M_w = 600.86; crystal size 0.31 × 0.11 × 0.10 mm; monoclinic; space group $P2_{1/n}$; a = 18.6106(6) Å, b = 9.3237(3) Å, c = 20.2857(6) Å; α = 90°, β = 98.9410(10)°, γ = 90°; V = 3477.20(19) Å^3; Z = 4; ρ_{calcd} = 1.148 g cm^{-3}; μ(Mo$_{K\alpha}$) = 0.071 cm^{-1}; T = 100(2) K; θ(min/max) = 2.984/26.372°; 38948 reflections collected; 6792 unique reflections (R_{int} = 0.0453); data/restraints/parameters: 6792/0/405; GoF on F^2 = 1.035; $R1$ = 0.0548 and $wR2$ = 0.1405; [5241 I>2σ(I)]; $R1$ = 0.0755 and wR = 0.1566 (all data); largest diff. peak and hole: 0.480 and –0.369 eÅ^3.

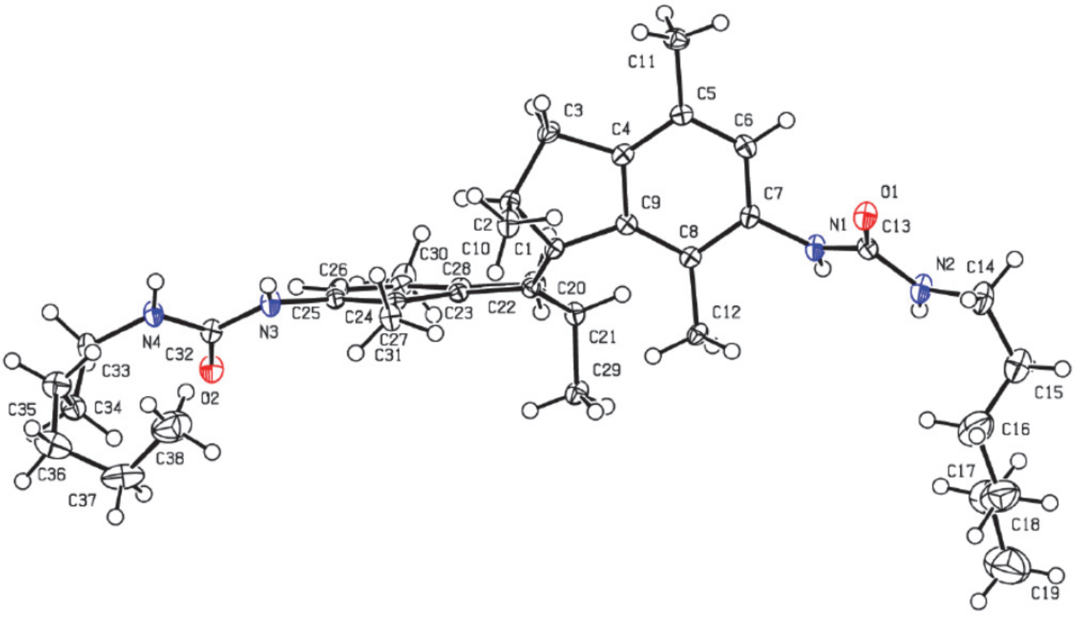

Fig. S15 Displacement ellipsoid plot at the 50% probability level of *trans*-**1b**.

DFT Calculations

The Gaussian 09 program[5] was used for geometry optimizations and the calculation of energies. Initially, different input geometries were optimized at the semi-empirical PM6 level to find the global minima. Further geometry optimizations were performed at the DFT B3LYP/6-31G+(d,p) level with tight convergence criteria and using an IEFPCM Toluene solvation model. The DFT geometries was found to have zero imaginary frequencies.

Table S1 Cartesian coordinates of *cis*-**1a**:

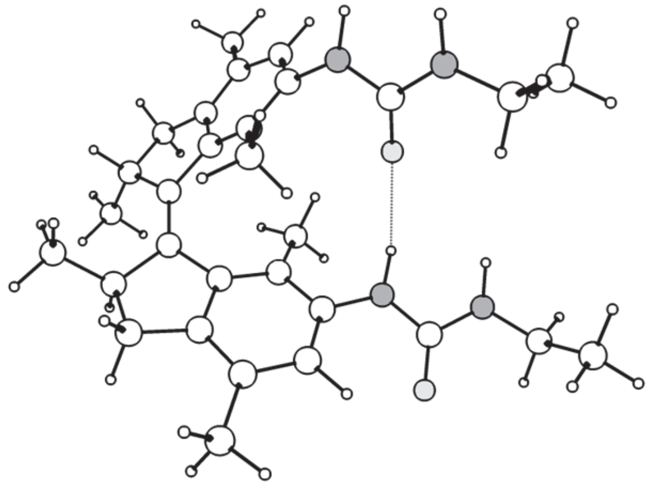

atom	X	Y	Z
C	3.118855000	0.088250000	-0.099927000
C	2.379644000	-1.176838000	0.115158000
C	3.048076000	-2.230695000	-0.541664000
C	4.305347000	-1.724604000	-1.193930000
C	4.532057000	-0.310216000	-0.577525000
C	1.242119000	1.829949000	-0.085345000
C	1.125258000	3.103025000	0.508130000
C	2.472296000	3.601257000	0.956327000
C	3.498703000	2.614184000	0.318621000
C	5.201156000	0.608997000	-1.608842000
C	1.265095000	-1.451407000	0.946916000
C	0.738151000	-2.756601000	0.878112000
C	1.363464000	-3.771322000	0.143181000
C	2.561649000	-3.540033000	-0.543348000
C	0.129666000	1.246102000	-0.734754000
C	-1.123829000	1.862182000	-0.526508000
C	-1.237695000	3.090510000	0.138322000
C	-0.104389000	3.756845000	0.620721000
C	4.681272000	2.395866000	1.273759000

C	2.644662000	1.370339000	-0.006214000
C	0.224159000	0.083452000	-1.692694000
C	-0.215789000	5.119345000	1.264234000
C	0.705257000	-0.473572000	1.946655000
C	3.278678000	-4.651037000	-1.273377000
N	-2.268753000	1.147355000	-0.962010000
N	-0.440594000	-3.088875000	1.615907000
C	-1.694767000	-2.569584000	1.324739000
N	-2.704175000	-3.065463000	2.108400000
C	-4.102812000	-2.687654000	1.933178000
O	-1.886299000	-1.716795000	0.448894000
C	-3.444077000	1.680132000	-1.452148000
N	-4.423991000	0.738711000	-1.682393000
O	-3.605687000	2.879721000	-1.698898000
C	-5.767057000	1.112689000	-2.108440000
C	-4.904128000	-3.689437000	1.095891000
C	-6.740531000	1.350624000	-0.948737000
H	4.171347000	-1.635856000	-2.282929000
H	5.164571000	-2.386970000	-1.038716000
H	5.200317000	-0.430510000	0.288457000
H	2.560660000	3.570485000	2.052995000
H	2.660112000	4.640006000	0.660401000
H	3.864548000	3.069189000	-0.613203000
H	4.529806000	0.782630000	-2.457405000
H	6.109918000	0.131025000	-1.993359000
H	5.491733000	1.581489000	-1.208824000
H	0.911554000	-4.760509000	0.124700000
H	-2.218585000	3.538367000	0.244703000
H	5.126794000	3.362123000	1.538111000
H	5.475294000	1.774769000	0.856543000
H	4.338784000	1.921238000	2.200418000
H	-0.428084000	0.272026000	-2.552395000
H	-0.086793000	-0.867799000	-1.248973000
H	1.246859000	-0.037700000	-2.053000000
H	-1.258547000	5.443151000	1.323530000
H	0.336152000	5.877075000	0.693770000
H	0.198018000	5.121614000	2.280043000
H	-0.185439000	0.042821000	1.578930000
H	1.446491000	0.286126000	2.200617000
H	0.418567000	-1.008095000	2.858140000
H	3.404236000	-4.418276000	-2.337616000
H	2.727886000	-5.593085000	-1.199201000
H	4.282722000	-4.817320000	-0.863488000
H	-2.236535000	0.148936000	-0.781207000
H	-0.417107000	-3.968035000	2.115486000
H	-2.478091000	-3.716151000	2.846649000
H	-4.112592000	-1.701840000	1.463496000
H	-4.548946000	-2.581041000	2.927953000
H	-4.283421000	-0.200518000	-1.336630000
H	-6.143816000	0.319398000	-2.763993000

H	-5.669654000	2.019312000	-2.709374000
H	-5.944292000	-3.358214000	0.999665000
H	-4.907773000	-4.681028000	1.561677000
H	-4.477997000	-3.784551000	0.092183000
H	-7.731533000	1.619291000	-1.332373000
H	-6.387343000	2.166084000	-0.310144000
H	-6.849508000	0.451314000	-0.331696000

Sum of electronic and zero-point Energies = –1536.278269

Gel-sol photoswitching experiment

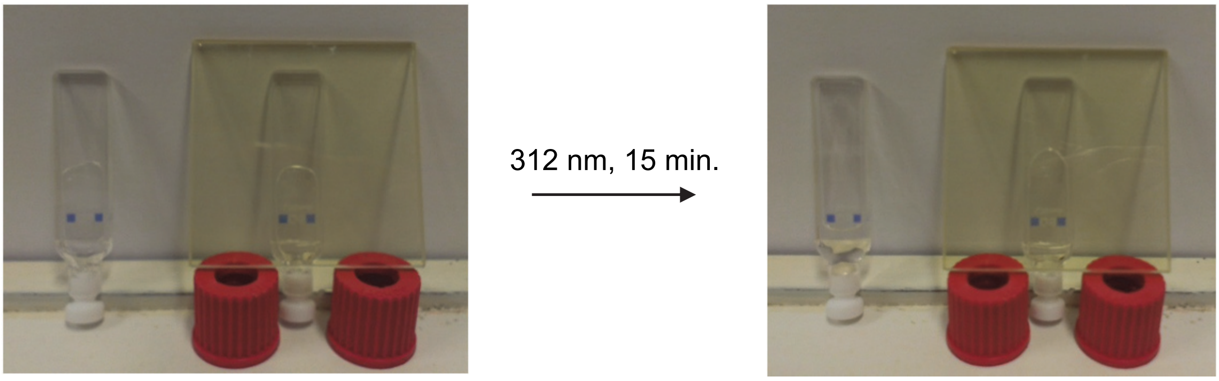

Fig. S16 Gel irradiation using 312 nm light with (right sample) and without (left sample) 420 nm cut-off filter. When the sample is shielded from light no gel-sol transition is observed.

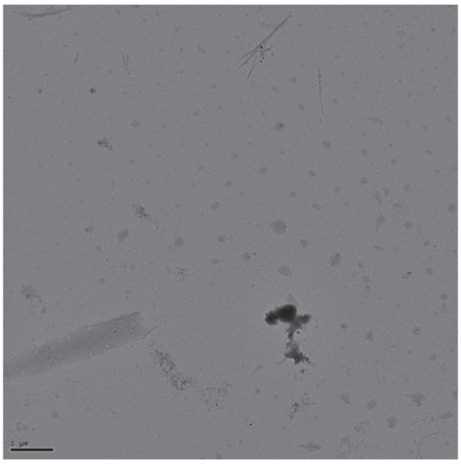

Fig. S17 Electron micrograph of a sample of the toluene gel (0.5 mg mL^{-1}) obtained with *trans*-**1b** after irradiation with 312 nm light giving a solution.

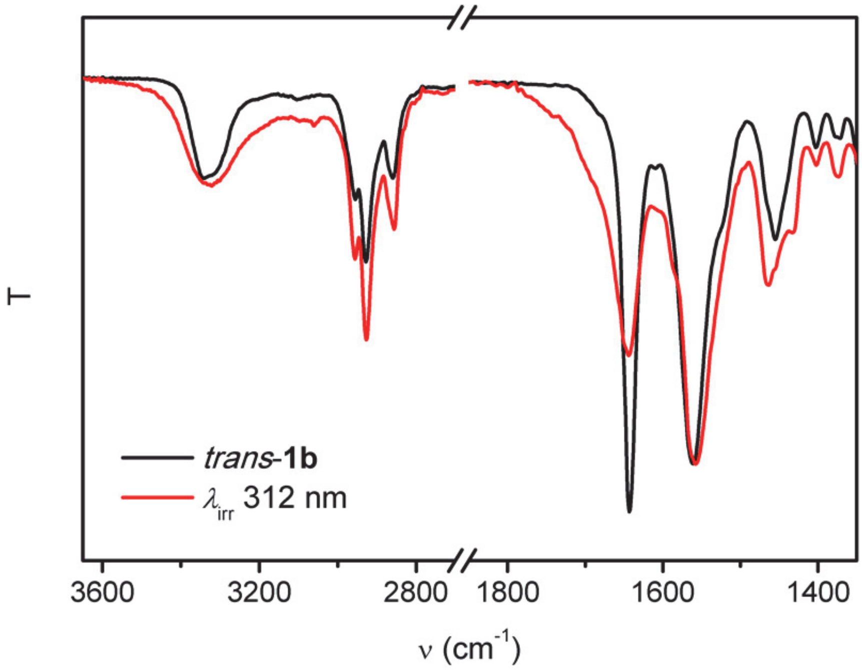

Fig. S18 Selected regions in the normalised IR spectra of solid *trans*-**1b** (black line) and a dried sample of the 312 nm light irradiated gel (red line) measured by FT-IR (ATR).

Notes and references

1 S. J. Wezenberg, M. Vlatković, J. C. M. Kistemaker and B. L. Feringa, *J. Am. Chem. Soc.*, 2014, **136**, 16784-16787.

2 Bruker, (2012). *APEX2* (v2012.4-3), *SAINT* (Version 8.18C) and *SADABS* (Version 2012/1). Bruker AXS Inc., Madison, Wisconsin, USA.

3 G. M. Sheldrick, *Acta Cryst.*, 2015, **A71**, 3-8.

4 G. M. Sheldrick, *Acta Cryst.*, 2008, **A64**, 112-122.

5 Gaussian 09, Revision D.01, M. J. Frisch, G. W. Trucks, H. B. Schlegel, G. E. Scuseria, M. A. Robb, J. R. Cheeseman, G. Scalmani, V. Barone, B. Mennucci, G. A. Petersson, H. Nakatsuji, M. Caricato, X. Li, H. P. Hratchian, A. F. Izmaylov, J. Bloino, G. Zheng, J. L. Sonnenberg, M. Hada, M. Ehara, K. Toyota, R. Fukuda, J. Hasegawa, M. Ishida, T. Nakajima, Y. Honda, O. Kitao, H. Nakai, T. Vreven, J. A. Montgomery, Jr., J. E. Peralta, F. Ogliaro, M. Bearpark, J. J. Heyd, E. Brothers, K. N. Kudin, V. N. Staroverov, T. Keith, R. Kobayashi, J. Normand, K. Raghavachari, A. Rendell, J. C. Burant, S. S. Iyengar, J. Tomasi, M. Cossi, N. Rega, J. M. Millam, M. Klene, J. E. Knox, J. B. Cross, V. Bakken, C. Adamo, J. Jaramillo, R. Gomperts, R. E. Stratmann, O. Yazyev, A. J. Austin, R. Cammi, C. Pomelli, J. W. Ochterski, R. L. Martin, K. Morokuma, V. G. Zakrzewski, G. A. Voth, P. Salvador, J. J. Dannenberg, S. Dapprich, A. D. Daniels, O. Farkas, J. B. Foresman, J. V. Ortiz, J. Cioslowski and D. J. Fox, Gaussian, Inc., Wallingford CT, **2013**.
